# Housing in Place: Housing, Neighbourhood and Resettlement for People from Refugee and Asylum Seeker Backgrounds in Australia

**DOI:** 10.1007/s12134-023-01008-w

**Published:** 2023-02-24

**Authors:** Anna Ziersch, Clemence Due, Moira Walsh

**Affiliations:** 1 College of Medicine and Public Health, Flinders University, Adelaide, Australia; 2 School of Psychology, University of Adelaide, Adelaide, Australia

**Keywords:** Refugee, Asylum seeker, Housing, Neighbourhood, Integration, Resettlement

## Abstract

Housing is an important part of building a new life for people from refugee and asylum seeker backgrounds. However, relatively little is known about how housing and neighbourhood experiences affect resettlement and integration. This paper explored experiences of housing and neighbourhood for refugees and asylum seekers in South Australia, Australia. A survey was completed by 423 participants, recruited through service providers, community networks and snowball sampling. Data was analysed using frequencies, chi-square analyses and multivariate logistic regression. The study identified aspects of housing and neighbourhood that were important to participants, as well as highlighting key problems. Housing satisfaction and neighbourhood satisfaction were positively associated, but housing satisfaction was lower than neighbourhood satisfaction. Both were significantly associated with overall satisfaction with life in Australia, although only neighbourhood satisfaction remained significant in the final multivariate model (alongside region of origin, visa and financial situation). Overall, the findings suggest that where housing is situated may be more important for resettlement satisfaction and integration than the housing itself. Policy and practice implications of the findings are discussed, alongside considerations for conceptualising integration.

## Introduction

Worldwide, there are more than 32 million refugees and almost 5 million asylum seekers in 2022, with only a fraction of refugees actually resettled (UNHCR, 2021).^1^ For newly arrived refugees and asylum seekers in receiving countries, finding good housing is a crucial first step to successful resettlement and an important part of the integration process, providing a place to establish oneself and develop community connections. Integration is a contested term but can be understood as a two-way process of accommodation between receiving and incoming communities (Ager & Strang, 2008; Ziersch, Miller, et al., 2020a, 2020b). Housing has been identified as a key aspect of integration (Ager & Strang, 2008), and attachment to place is important for those who have experienced forced migration (Farmer, 2021; Sampson & Gifford, 2010). However, there is relatively little empirical research on the impact of housing and neighbourhood experiences on resettlement experiences, particularly for asylum seekers. This paper explores the housing and neighbourhood experiences of people from refugee and asylum seeker backgrounds in South Australia drawing on survey data from 423 participants and examines the impact of these experiences on overall resettlement satisfaction. In doing so, it also seeks to further explore the relevance of both housing and neighbourhood for theoretical conceptualisations of integration.

### Background

Refugees and asylum seekers are typically a marginalised group of the global community, many of whom have experienced violence, trauma and dislocation (Fazel et al., 2005; Hollifield et al., 2002). Integration and positive outcomes post-resettlement rely on a range of factors including access to education, appropriate employment, social connections, services, stable migration status and safety from violence and harm, as well as suitable housing (Ager & Strang, 2008; Phillimore & Goodson, 2008). Ager & Strang’s (2008) model of integration has been influential in migration studies, outlining ten indicators of integration, organised into four domains. The first domain ‘Foundation’ deals with rights and citizenship; ‘Facilitators’ includes language and cultural knowledge, and safety and stability; ‘Social Connection’ draws on Putnam’s dimensions of social capital (Putnam, 2000)—social bonds (within own community), bridges (to other communities) and linkages (to wider social institutions). The last domain ‘Means and Markers’ includes employment, education and health as well as housing, which through their interaction with other elements of integration are seen both as indicators of successful integration as well as an avenue to integration. In this way, securing appropriate housing can help facilitate other aspects of integration, while access to suitable housing is also a positive indicator of integration in its own right.

While Ager and Strang highlight elements of neighbourhood in their description of housing, and the importance of contexts for integration more generally are noted in this and later incarnations of the model (Ndofor-Tah et al., 2019), neighbourhood does not explicitly feature. This is also true of other influential integration models. For example, Berry’s Acculturation Model sees integration as one outcome of migration (other examples are assimilation, separation and marginalisation) where an individual adopts receiving cultural norms whilst also maintaining heritage culture (Berry, 1997). However, Berry’s model is a psychological one, and thus is focused even more on individuals and less on contexts. The Holistic Integration Model developed by Hynie and colleagues (Hynie et al., 2016) builds on Ager and Strang’s model by highlighting the interdependence of the various aspects of integration, the potential influence of prior social location and experiences on refugee integration, and the effects of the social, economic and political/ideological setting of receiving communities. Importantly, they suggest that structural and socio-cultural elements have a bearing on the functional aspects of integration and in creating a feeling of welcome, which affects how refugees navigate their relationships and roles in their new country, their day-to-day lives and the process of integration at the social/subjective and functional levels. While this model touches on the importance of welcoming and safe neighbourhoods for refugee integration, there remains gaps in knowledge and current integration models concerning the role of neighbourhood and related contextual elements. As such, in this paper, we draw on empirical data to examine housing and neighbourhood experiences and consider the implications for expanding these models of integration.

#### Housing, Neighbourhood and Resettlement

Housing and home are both material and symbolic spaces, representing physical shelter and also symbolic resources such as status, identity, privacy and autonomy (Easthope, 2004; Hiscock et al., 2001; Mallett, 2004). Housing is also particularly important for ontological security (a sense of identity and constancy in relation to self, social and physical environments (Giddens, 1991)), which has particular relevance for refugees and asylum seekers given the likelihood that they have previously experienced heightened levels of threat in a range of situations (Carter et al., 2009; Easthope et al., 2018; Fozdar & Hartley, 2014; Phillips, 2006).

In many countries of resettlement—including Australia—asylum seekers and refugees are provided with some housing in initial stages. However, emerging research indicates that many face difficulties securing appropriate, affordable and secure housing once this support has ended, or in cases where it is not offered at all. In Australia and other similar resettlement countries such as Canada, the UK, Germany and New Zealand, research concerning the housing experiences of refugees has identified issues including the cost of private rental housing; insecure tenure; lack of access to public housing; language barriers; challenging application processes including the need for references; requirements to be employed; low income levels; discrimination; lack of appropriate housing stock especially for large families; overcrowding; and homelessness (see Ziersch & Due, 2018).

Importantly, asylum seekers can face housing-related difficulties, above and beyond those experienced by refugees. For example, studies in Australia (Ziersch et al., 2017a, 2017b and Canada (Murdie, 2008) found issues in securing housing for asylum seekers who were less likely to have the required paperwork for rental accommodation, had less eligibility for welfare and had difficulties signing leases due to short term visas. Asylum seekers may also be reluctant to raise housing issues with landlords due to fear of repercussions for visa applications (Ziersch et al., 2017a, 2017b). Limited government supports and restrictions on work rights have also been found to increase risk of homelessness for asylum seekers in the UK (Phillips, 2006; Robinson & Reeve, 2006).

While housing issues affect resettlement and other elements of integration, research also suggests that they cannot be separated from the neighbourhood context (Netto, 2011; Rose & Ray, 2001). Place attachment is an important aspect of building a new life for people from refugee and asylum-seeking backgrounds (Farmer, 2021; Sampson & Gifford, 2010). Inclusive neighbourhoods that help develop social networks, a sense of belonging and access to appropriate services and resources have been found to promote integration for refugees and asylum seekers (Ager & Strang, 2008; Carter et al., 2009; Hickman et al., 2008; Platts-Fowler & Robinson, 2015; Spicer, 2008).

However, research focusing on the neighbourhood experiences of refugees and asylum seekers has also highlighted a range of potential difficulties that may undermine integration. For example, while Easthope and colleagues (Easthope et al., 2018) have argued that living in so-called gateway suburbs may assist with integration through the formation of networks and the development of social capital, other research indicates that the initial suburbs refugees and asylum seekers are often placed in can lead to a range of problems that negatively affect integration. These suburbs typically have low-cost housing and often low access to services (Beer & Foley, 2003; Easthope et al., 2018; Flatau et al., 2015; Griffiths et al., 2006; Phillips, 2006; Rose & Ray, 2001; Stewart, 2012). Additionally, Easthope notes that while post-World War Two settlement was characterised by supportive immigration and social policies that meant the people in such neighbourhoods could form grass-roots support and community organisations to invigorate suburbs, such policies no longer exist in many countries of resettlement. The result is that gateway suburbs are generally characterised by economic disadvantage, can have higher levels of crime and disorder and may lack essential services (Carter et al., 2009; Phillimore & Goodson, 2006; Phillips, 2006; Spicer, 2008). Refugees and asylum seekers also face challenges building bridging ties within neighbourhoods, particularly socio-economically disadvantaged neighbourhoods, when they are ‘visibly different’, have different cultural expectations about neighbourliness and have limited language skills (Beer & Foley, 2003; Colic-Peisker & Tilbury, 2008; Flatau et al., 2014). As such, neighbourhoods can be sites of exclusion for refugees and asylum seekers (Beer & Foley, 2003; Guerin et al., 2013; Hebbani et al., 2017; Huizinga & van Hoven, 2018; Phillips, 2006; Ziersch et al., 2020a). In addition, refugees are often highly mobile, partly as a result of housing and neighbourhood issues as well as relocation to be closer to ethnic communities (Beer & Foley, 2003; Fozdar & Hartley, 2014; Harte et al., 2009; Netto, 2011; Phillips, 2006).

While housing issues for refugees, and to some extent asylum seekers, have been relatively well outlined—and there is also a growing evidence base about neighbourhood experiences—there is relatively little quantitative research examining housing and neighbourhood impacts on resettlement satisfaction or differences in demographic features such as visa type or region of origin—nor what this might mean for considerations of integration. As such, drawing on survey responses from 423 participants, this paper examines the housing and neighbourhood experiences of refugees and asylum seekers in South Australia and aimed to address the following research questions: (1) What are the key housing and neighbourhood issues and are there socio demographic differences in these; (2) what is the relationship between housing experiences, neighbourhood experiences and overall resettlement satisfaction? In exploring these research questions, we also aimed to consider the implications of the findings for theoretical conceptualisations of integration.

### Study Context

Over the last 10 years, Australia has resettled over 170,000 refugees (Refugee Council of Australia, 2017). In 2018–2019, 55% of all offshore visas (granted to those living outside Australia) were granted to persons born in the Middle East, 23% to persons born in Asia and 22% to persons born in Africa (Australian Government, 2019). In addition to refugees resettled through the ‘offshore’ program, those who claim asylum once in Australia receive a temporary ‘bridging’ visa and since 2014, even if these claimants are subsequently found to be a refugee, they are only eligible for a temporary visa of 3–5 years, after which they may apply again. These temporary visa holders have varying entitlements to work, length of stay, eligibility for welfare and other government assistance; however, there is overall limited resettlement support as well as restrictions on travel and services and no access to family reunion (Reilly, 2016).

As with many other countries of resettlement, Australia—as part of an overall resettlement support program—provides some housing support to refugees and asylum seekers. However, this is largely temporary and often dependent on visa status, with greater protections for those on permanent visas than those on temporary visas. At the time this research was conducted (2015–2016), permanent visa holders were provided with housing for 6 months at a subsidised rate, while asylum seekers were only eligible for four weeks. After this time, people were expected to seek alternate housing, generally in the private rental market given the contracting social housing stock and the removal of refugee status as a priority category in South Australia. Since then, the period of initial housing has been shorter (generally one month), with assistance from housing providers to find ongoing housing earlier in the settlement process.

South Australia generally resettles approximately 10% of the refugees accepted through the UNHCR program, with the majority of new arrivals being resettled in Adelaide (Laukova et al., 2022). South Australia is the southern central state of mainland Australia. It has a population of 1.8 million people, with 80% of these living in the capital, Adelaide, the fifth largest city in Australia (Government of South Australia, 2022). In Adelaide, residential development has been lineal rather than radial, with a small CBD ringed by parklands, extending around 40 km north of the CBD and around 35 km south (Government of South Australia, 2008). Housing is generally detached and low density. While there is no specific housing dispersal scheme, there is clustering of refugee communities in areas with affordable housing and access to services. In Adelaide, this is generally in the outer suburbs to the north of the city. It is noted that this research occurred prior to the COVID-19 pandemic. Anecdotal reports from service providers suggest that the pandemic and associated social and economic impacts have significantly exacerbated housing difficulties for refugees and asylum seekers.

## Materials and Methods

Ethics approval was obtained from the Flinders University (then) Social and Behavioural Research Ethics Committee and the researchers paid particular attention to potential issues of coercion and informed consent, and power imbalances between researchers and participants, as well as concerns about confidentiality and anonymity (Block et al., 2013; Ziersch et al., 2017c).

As part of a broader project examining housing, social inclusion and health and well-being, a survey was completed by refugees and asylum seekers aged 18 and above, living in Australia for 7 years or less, currently resident in any part of Adelaide, South Australia. Survey participants (*N* = 423) were recruited in a range of ways to ensure broad participation including through non-government organisations, community groups and passive snowball sampling. An information section accompanied the survey outlining key ethical considerations and completion of the survey was taken as an indication of consent. Data collection occurred between June 2015 and June 2016.

The project was conducted in partnership with a project reference group and a refugee and asylum seeker advisory group who co-developed and helped pilot the survey questions. The final survey included closed- and open-ended questions about housing and neighbourhood experiences, alongside other aspects of resettlement. The survey and project documentation were translated into five key languages (Arabic, Dari, Farsi, Nepali and Swahili) and could be completed in hard copy or online (English only). Survey items included the following.

*Demographic questions* included gender (male/female); age (18–29, 30–49, 50 +); country of birth (open ended, categorised for analysis to region of origin: Middle East, Africa, South East Asia); time in Australia (< 6 months, 7 months to < 2 years, 2 years to < 5 years, 5 + years); current visa (dichotomised as permanent or temporary), financial satisfaction (5-point Likert scale from very unhappy to very happy, dichotomised for analysis as happy/very happy or neutral/unhappy/very unhappy) and employment (later dichotomised as employed or other).

*Housing and neighbourhood related questions* including questions about housing in Australia (current type, number of moves, number of bedrooms, how they found their current house) as well as plans for housing in the next 6 to 12 months. In further analysis, three of these variables were used: (1) housing type (simplified to public/community, private rental, owner occupied and other); (2) time in housing (< 6 months, > 6 months to < 2 years, > 2 years to < 3 years, > 3 years) and; (3) whether housing was ‘crowded’ or not (defined as more than 2 people per bedroom (Australian Institute for Health & Welfare, 2020).

The survey also asked about a range of housing and neighbourhood experiences and preferences, with responses developed in partnership with the advisory groups and based on previous research (Beer & Foley, 2003; Flatau et al., 2014; Forrest et al., 2013; Fozdar & Hartley, 2014). Questions included the following: What was important when choosing housing (10 options), problems with current housing (24 options), what was important in neighbourhoods (12 options), and problems with current neighbourhood (11 items). Specific responses to each are provided in the ‘Results’ section. Housing and neighbourhood satisfaction were measured with the questions: ‘Overall, how do you feel about your current housing?’ and ‘Overall, how do you feel about your current neighbourhood’, with a 5-point visual ‘smiley face’ Likert scale ranging from very unhappy to very happy (dichotomised for analysis to very happy/happy or neutral/unhappy/very unhappy).

Neighbourhood disadvantage was measured by assigning to suburbs of residence deciles of disadvantage drawn from the 2016 Census Australian Bureau of Statistics Index of Relative Socioeconomic Disadvantage (Australian Bureau of Statistics, 2018). This area level index is a combination of several indicators such as income, unemployment, occupational level and education. For further analysis, deciles were dichotomised into those who were in the two highest deciles for disadvantage (higher disadvantage) and those in the remaining deciles (lower disadvantage).

*Overall resettlement satisfaction* was assessed with the question ‘Overall, how happy are you with your life in Australia?’, again using the visual Likert scale. This question reflects a similar question in the Building a New Life in Australia longitudinal study of refugees (2013–2018), to encompass overall experiences of resettlement, as a proxy for integration.

Analyses were conducted using SPSS version 27 and included frequencies to provide an overall picture of housing and neighbourhood experiences, and *t*-tests and chi-square tests as well as a multivariate logistic regression to examine the relationships between demographic variables, housing and neighbourhood satisfaction and overall resettlement satisfaction.

## Results

### Participant Characteristics

The participants were relatively evenly split by gender and were largely under 49 years of age (Table 1). Over half came from the Middle East, reflecting the recent focus of the humanitarian program in Australia, and over 70% were on a permanent refugee visa. More than 60% had been in Australia for 2 or more years. Only 13% were currently employed and over 70% were not happy with their financial situation.

**Table 1:** Participants

	*N* (%)
Gender Female Male	(20 missing) 188 (46.7) 215 (53.3)
Age 18–29 30–49 50 +	(4 missing) 173 (41.3) 202 (47.8) 44 (10.4)
Region Middle East Africa South East Asia	(8 missing) 221 (53.3) 137 (33.0) 57 (13.5)
Visa Permanent Temporary	(14 missing) 296 (72.4) 113 (27.6)
Time in Australia 6 months or less 7 months– < 2 years 2– < 5 years 5 + years	(2 missing) 62 (14.7) 103 (24.5) 190 (45.1) 66 (15.7)
Employment Employed Non-employed	(21 missing) 54 (13.4) 348 (86.6%)
Financial satisfaction Satisfied Not satisfied/neutral	(36 missing) 111 (28.7) 276 (71.3)

### Housing Experiences in Australia

All participants were currently living in South Australia, with only 29 people (7%) having lived in another state of Australia. Over two-thirds (67%) had lived in more than one house since living in Australia, including their initial allocated housing, with a mean of 2.34 houses (range 1–9, *SD* = 1.22).

As seen in Table 2, most participants (60%) were currently living in private rental accommodation, with 18% living in housing provided through the main housing provider for newly arrived refugees, and a small number living in other forms of housing. Most people had been living in their current house for 2 years or less. The number of people living in the house ranged from 1 to 13 (*M* = 4.55, *SD* = 2.287). Reflecting the main housing stock in Adelaide, the most common number of bedrooms in the house was three. Fifty-one participants (13.1%) lived in housing considered crowded.

**Table 2:** Housing in Australia

Current housing type Not for profit main provider Private rental Own house/mortgage Public housing (community and housing trust) Other	*N* (%) 72 (17.9) 242 (60.0) 14 (3.5) 45 (11.2) 30 (7.4)
Time in current house Less than 6 months 6 months– < 2 years 2 years– < 3 years 3 + years	*N* (%) 127 (34.1%) 158 (42.1%) 65 (17.5%) 25 (6.7%)
Number of bedrooms 0 1 2 3 4 5 6	*N* (%) 1 (0.2%) 23 (5.6%0 106 (25.8%) 198 (48.2%) 63 (15.3%) 11 (2.7%) 9 (2.2%)
Crowding (3 + people per bedroom)	
Crowded Not crowded	339 (87.9%) 52 (13.1%)

Most people found their current house through family and friends (*N* = 118, 28.3%), followed by their case worker (*N* = 97, 23.3%), on their own (*N* = 95, 22.8%), through a real estate agent (*N* = 80, 19.2%), other service provider (*N* = 25, 6.0%) or other way (*N* = 10, 2.4%), with some using multiple sources of support).

Affordable rent, safety and good condition were the top three factors identified as important in housing (Table 3), followed by good neighbourhood and then other housing features. Home ownership was rated the lowest.

**Table 3:** What is important in housing

	*N*	Valid %
Affordable rent	293	71.3
Housing in which you feel safe	250	60.8
In good condition	243	59.1
Good neighbourhood	233	56.7
Enough bedrooms	231	56.2
Enough living areas	182	44.3
Enough bathrooms	171	41.6
A garden/yard	151	36.8
Housing you are buying or own	83	20.2
Other	22	5.5

^*^12 missing

Over three quarters (*N* = 309, 77.4%) of participants identified at least one problem with their current housing, with the total number of problems ranging from 0 to 20 (*M* = 2.49, *SD* = 2.61). Given that most people were not living in their first house, this suggests that some people continue to encounter housing challenges in subsequent housing. Issues were identified as relevant to either finding housing or to housing once secured, with communicating in English a key issue identified by participants that is relevant to both these. As seen in Table 4, key issues finding housing included getting to open inspections, a lack of references or rental history and affordable housing not being available in the neighbourhoods where participants wanted to live. Once secured, the most common housing issues were the cost of rent, issues with heating and cooling, not enough bedrooms and bathrooms and living areas.

**Table 4:** Problems with current housing

	*N*	Valid %
Rent too expensive	108	26.5
Heating and cooling	96	23.6
Not enough bedrooms	78	19.1
Not enough bathrooms	64	15.7
Not enough living areas	60	14.7
Housing not in good condition	56	13.8
Communicating in English	56	13.7
Getting things fixed	49	12.0
Lack of affordable housing in area I want to live in	48	11.8
Getting to open inspections	41	9.7
Applying for public housing	39	9.6
Understanding my rights and responsibilities	36	8.8
No referees/rental history	37	8.7
Looking after house and garden	34	8.3
Housing too crowded	32	7.9
Problems with interpreters	29	7.1
Difficulties with neighbours	27	6.6
Housing not safe	25	6.1
Discrimination	19	4.7
Period inspections	19	4.7
Problems with real estate agents or landlords	17	4.2
Getting a mortgage	17	4.2
Securing housing for large family	16	3.9
Other problem	15	3.7
Getting bond returned	10	2.5

^*^15 missing

When asked about housing plans in the next 6–12 months, 34.2% (*N* = 137) were planning to stay in their current housing. The remainder did not know their plans (*N* = 106, 26.4%), or were planning on moving to other rental housing (*N* = 79, 19.7%) or other housing of another type (*N* = 67, 16.6%).

#### Housing Satisfaction

Just over half reported being happy or very happy (*N* = 319, 52.9%) with their current housing, with the remainder (*N* = 195, 47.1%) neutral, unhappy or very unhappy.

Table 5 indicates that region, visa type and financial satisfaction were significantly associated with housing satisfaction. Participants from Southeast (SE) Asia were most satisfied with their housing, followed by those from Africa and then those from the Middle East. Those on permanent visas were more satisfied with their housing than those on temporary visas. Participants who were struggling financially were more likely to be unsatisfied with their housing than those who felt greater financial security. Gender, age and time in Australia were not significantly associated with satisfaction, and neither were any of the current housing specific variables (type, time in housing and crowding).

**Table 5:** Current housing satisfaction by demographic variables

	Happy	Not happy
Gender		
* Male* * Female*	97 (48%) 112 (54%)	88 (52%) 97 (46%)
Age		
* 18–29 years* * 30–49 years* * 50* + *years*	91 (55%) 106 (53%) 21 (50%)	76 (45%) 95 (47%) 21 (50%)
Region ***		
* Middle East* * Africa* * SE Asia*	96 (44%) 74 (56%) 42 (74%)	120 (56%) 59 (44%) 15 (26%)
Time in Australia		
> *6 months* * 7 months to* < *2 years* * 2–5 years* > *5 years*	32(52.5%) 57 (55.3%) 94 (50.8%) 34 (54.0%)	29 (47.5) 46 (44.7%) 91 (49.2%) 29 (46.0%)
Visa type**		
* Permanent* * Temporary*	167 (57%) 46 (42%)	125 (43%) 63 (58%)
Employment		
* Employed* * Not employed*	33 (62.3%) 178 (51.9%)	20 (37.7%) 165 (48.1%)
Financial satisfaction***		
* Happy* * Not happy*	90 (82%) 113 (42%)	20 (18%) 159 (59%)
Housing type		
* Community/public* * Private rental* * Owner occupied* * Other*	65 (56%) 120 (51%) 10 (83%) 13 (42%)	51 (44%) 117 (49%) 2 (17%) 18 (58%)
Time in housing		
* Up to 6 months* > *6 months–* < *2 years* * 2 years* * 3* +	71 (56%) 75 (48%) 30 (48%) 16 (67%)	55 (44%) 81 (52%) 33 (52%) 8 (33%)
Time in housing		
* Not crowded* * Crowded*	181 (54%) 26 (50%)	153 (46%) 26 (50%)

^*^p < .05; **p < .01; ***p < .001

We also looked at the extent to which experiencing housing problems was associated with housing satisfaction and found a significant association (*t* =  − 3.673, *df* = 389, *p* < 0.000). Those who were happy with their housing had fewer problems (*M* = 1.77, *SD* = 2.09) compared to those who were not happy (*M* = 3.34 problems, *SD* = 2.88).

### Neighbourhood Experiences in Australia

The main feature identified as important when choosing a neighbourhood was safety, followed by friendliness and good neighbours, and then access to amenities such as shops, schools and childcare, public transport and local services (Table 6).

**Table 6:** What is important in a neighbourhood

	*N*	Valid %
Feeling safe	319	77.1
Friendly	233	56.3
Good neighbours	212	51.2
Good public transport	207	50.0
Close to schools/childcare	198	47.8
Close to shops	173	41.9
Good local services	171	41.3
Close to relatives/family	138	33.3
Close to place of worship	132	32.0
Close to friends	128	30.9
Close to work	95	22.9
Other	17	0.8

In relation to problems with current neighbourhood, 44% (*N* = 197) reported at least one problem. The most commonly identified problems were distance from social connections and services as well as issues with safety and neighbourhood relations (Table 7).

**Table 7:** Problems in current neighbourhood

	***N***	**%**
Too far from friends	52	15.0%
Too far from schools/childcare	49	14.1%
Too far from place of worship	47	13.5%
Neighbourhood not safe	40	11.5%
Neighbourhood not friendly	38	11.0%
Too far from family/relatives	33	9.5%
Too far from shops	28	8.1%
Trouble with neighbours	21	6.1%
Too far from public transport	17	4.9%
Other	12	3.5%
Too far from work	10	2.9%
	347	100

Table 8 shows the deciles of disadvantage (Australian Bureau of Statistics, 2018) for neighbourhood suburbs. Over half the participants lived in suburbs in the two highest deciles of disadvantage.

**Table 8:** Suburb IRSD decile of disadvantage

Decile	*N*	%
1	153	36.2
2	62	14.7
3	53	12.5
4	26	6.1
5	29	6.9
6	17	4.0
7	21	5.0
8	12	2.8
9	15	3.5
10	3	.7
Missing	32	7.6
Total	423	100

Rates of satisfaction with current neighbourhood were higher than those for housing, with over two-thirds happy (*N* = 275, 66.9%) and the remainder neutral, unhappy or very unhappy (*N* = 136, 33%).

As with housing, region, visa and financial situation were related to neighbourhood satisfaction, with those from SE Asia again the happiest, and those on temporary visas and those struggling financially less satisfied with their neighbourhood (Table 9).

**Table 9:** Current neighbourhood satisfaction by demographic variables

	Happy	Not happy
Gender		
*Male* *Female*	115 (63.2%) 146 (69.5%)	67 (36.8%) 64 (30.5%)
Age		
*18–29 years* *30–49 years* *50* + *years*	115 (68.9%) 132 (66.0%) 27 (67.5%)	52 (31.1%) 68 (32.0%) 13 (32.5%)
Region **		
*Middle East* *Africa* *SE Asia*	137 (63.4%) 86 (65.2%) 48 (85.7%)	79 (36.6%) 46 (32.8%) 8 (14.3%)
Time in Australia		
> *6 months* *7 months to* < *2 years* *2–5 years* > *5 years*	39 (65.0%) 63 (61.8%) 127 (67.9%) 45 (73.8%)	21 (35.0%) 39 (38.2%) 60 (32.1%) 16 (26.2%)
Visa type **		
*Permanent* *Temporary*	209 (72.0%) 62 (56.9%)	81 (28.0%) 47 (43.1%)
Employment		
*Employed* *Not employed*	32 (61.5%) 235 (68.5%)	20 (38.5%) 108 (31.5%)
Financial satisfaction ***		
*Happy* *Not happy*	92 (82.9%) 165 (61.1%)	19 (17.1%) 105 (38.9%)

Neighbourhood satisfaction was significantly associated with the number of neighbourhood problems—those who were satisfied had an average of 0.62 problems and those who were not satisfied reported 1.61 problems (*t* = 6.792, *d*f = 388, *p* =  < 0.001). Neighbourhood satisfaction was not significantly associated with neighbourhood disadvantage, with 66.9% (117/175) of those living in the less disadvantaged suburbs satisfied with their neighbourhood, compared to 67.7% (114/213) of those living in the more disadvantaged area (*χ*^2^ = 0.024, *df* = 1, *p* = 0.876).

We also examined the relationship between neighbourhood and housing satisfaction and found a significant association (*χ*^2^ = 55.561, *df* = 1, *p* < 0.000). Almost three quarters of people (*N* = 100, 73.5%) who were not satisfied with their neighbourhood were also not satisfied with their housing. Likewise, two-thirds (*N* = 179, 65.6%) of those who were satisfied with their neighbourhood were also satisfied with their housing.

### Overall Resettlement Satisfaction

More than three quarters (*N* = 296, 75.7%) of participants were happy/very happy with their life in Australia compared to *N* = 95 (24.3%) who were not.

We examined the role of housing and neighbourhood satisfaction in predicting overall resettlement satisfaction in a multivariate logistic regression, alongside other key demographic variables (Table 10). In the first model, housing satisfaction significantly predicted overall resettlement satisfaction, alongside region, visa status and financial status. Housing satisfaction was associated with a twofold greater chance of being satisfied overall with resettlement (OR = 1.992, 95% CI = 1.085–3.656). Compared to those from SE Asia, those from Africa were less likely to be satisfied with life in Australia (OR = 0.155, 95% CI = 0.045–0.536), with no difference for those from the Middle East. Permanent visa holders were greater than seven times more likely than temporary visa holders to be satisfied with overall resettlement (OR = 7.234, 95% CI = 3.341–15.666). Those who were happy with their financial situation were nearly four times as likely to also be happy with their overall resettlement experiences (OR = 3.989, 95% CI = 1.556–10.224). The model was significant (*χ*^2^ = 74.127, df = 12, *p* = 0.000) and explained 30% of the variance (Table 10).

**Table 10:** Logistic regression for resettlement satisfaction

	Model 1			Model 2		
	*Odds ratio*	*SE*	*95% CI*	*Odds ratio*	*SE*	*95% CI*
Gender**—**female	1.203	0.299	0.669**–**2.163	1.279	0.306	0.702**–**2.329
Age (50 + years)						
18–29 years	0.281	0.634	0.081**–**0.975	0.276	0.653	0.077**–**0.993
30–49 years	0.344	0.611	0.104**–**1.139	0.341	0.627	0.100**–**1.166
Region (SE Asia)						
Middle East	0.547	0.567	0.180**–**1.662	0.657	0.567	0.216**–**1.995
Africa	0.155**	0.634	0.045**–**0.538	0.186**	0.630	0.054**–**0.640
Time in Australia (5 + years)						
6 months or less	0.330	0.654	0.091**–**1.189	0.429	0.658	0.118**–**1.560
7 months– < 2 years	0.442	0.583	0.141**–**1.387	0.614	0.597	0.190**–**1.980
2– < 5 years	0.661	0.559	0.221**–**1.976	0.778	0.563	0.258**–**2.347
Visa**—**permanent	7.234***	0.394	3.341**–**15.666	6.927***	0.399	3.169**–**15.143
Employment**—**employed	0.921	0.475	0.363**–**2.238	1.154	0.497	0.436**–**3.056
Financial satisfaction**—**satisfied	3.989**	0.480	1.556**–**10.224	3.821**	0.481	1.489**–**9.803
Housing satisfaction**—**satisfied	1.992*	0.310	1.085**–**3.656	1.511	0.326	0.797**–**2.863
Neighbourhood satisfaction**—**satisfied				2.522**	0.324	1.336**–**4.758
Constant	5.992			2.657		
Nagelkerke R squared	0.303			0.336		

^*^p < .05; **p < .01; ***p < .001

When neighbourhood satisfaction was added into the second model (Table 10), region, visa status and financial satisfaction remained significant, but housing satisfaction did not. Those who were satisfied with the neighbourhood that they lived in were more than 2.5 times as likely to report resettlement satisfaction (odds ratio = 2.522, 95% CI = 1.336–4.758). The model was significant (*χ*^2^ = 82.295, df = 13, *p* = 0.000) and explained a third of the variance.

## Discussion

Overall, this study of refugee and asylum seeker experiences in South Australia indicates that housing and neighbourhood are important elements of resettlement, highlighting some of the housing and neighbourhood factors most valued by this cohort as well as the challenges they face. Importantly, housing and neighbourhood satisfaction were both significantly associated with overall resettlement satisfaction at a bivariate level. However, once included in a multivariate model, only neighbourhood satisfaction remained significant, alongside a number of demographic features (region, visa and financial situation). This suggests that where housing is situated may be more important for resettlement satisfaction than features of the housing itself. We discuss these findings below, as well as examining implications for theoretical conceptualisations of integration and also policy and practice.

### Survey Findings

Reflecting the Australian literature, most of the samples were living in private rental housing, with a small number in housing provided by a non-government provider as part of their initial government-provided resettlement support (Flatau et al., 2014). Results indicated high mobility as found in other studies of refugee housing careers (Beer & Foley, 2003; Carter et al., 2009; Harte et al., 2009; Phillips, 2006). Some of this mobility may relate to issues with current housing (or neighbourhood) or period of settlement housing support but may also reflect the desire to move to be closer to social networks or other resources (Spicer, 2008).

In terms of what was important in housing and neighbourhoods, affordable rent was the top ranked factor, while safety featured highly in terms of both housing and neighbourhood, reflecting previous research particularly in relation to ontological security (Carter et al., 2009; Easthope et al., 2018; Fozdar & Hartley, 2014; Phillips, 2006; Ziersch et al., 2017a, 2017b). High on the list for good housing was also a good neighbourhood, highlighting the interconnectedness of housing and neighbourhood experiences, as found in other research (Fozdar & Hartley, 2014). Participants reported a range of issues with their current housing, reflecting the broader Australian and international literature that highlights multiple barriers for new arrivals to accessing housing, and issues with housing once it is secured. Some of these issues reflect similar concerns experienced by many in the broader population with limited income (e.g., high rents, lack of heating and cooling). However, for new arrivals, these issues can compound other settlement stressors.

Overall, less people reported an issue with their neighbourhoods than their housing. The main neighbourhood issues related to location in terms of distance from friends and family and services, as well as issues with safety and neighbourhood relations. This reflects previous literature concerning neighbourhood experiences (Beer & Foley, 2003; Carter et al., 2009; Guerin et al., 2013; Hebbani et al., 2017; Huizinga & van Hoven, 2018; Phillips, 2006; Ziersch et al., 2017a, 2017b), which has identified potentially different cultural expectations about neighbourliness as well as experiences of racism and discrimination as potential factors at play in relation to neighbourhood safety and neighbour relations.

The high rate of people from refugee and asylum seeker backgrounds living in the most disadvantaged areas may relate to the limited incomes many have available for rent, leading them to live in areas with more affordable housing (Beer & Foley, 2003; Easthope et al., 2018; Phillips, 2006; Rose & Ray, 2001; Stewart, 2012; Ziersch et al., 2017a, 2017b). Interestingly, neighbourhood disadvantage was not significantly associated with neighbourhood satisfaction suggesting that other neighbourhood features were more important. This supports Easthope and colleagues’ argument about potential benefits of ‘gateway’ suburbs deemed ‘disadvantaged’ (Easthope et al., 2018), in terms of other neighbourhood features that may be valued such as building social networks and proximity to services supporting refugees.

Overall levels of satisfaction with housing and neighbourhood were relatively high. As Rose & Ray, (2001) note, it is difficult to know whether the reference point for satisfaction with current housing is other housing (or neighbourhood) in Australia or housing (or neighbourhood) in a country of origin or in other countries of transit or refuge. Moreover, low expectations and the absence of alternatives may cause refugees and asylum seekers to indicate that they are satisfied with unsuitable accommodation (Phillips, 2006). Reflecting the findings above, participants in this study were more satisfied and indicated fewer problems with their neighbourhoods than their housing. This suggests that people may have been prepared to compromise on housing suitability and quality in order to be in their preferred neighbourhood and, as noted above, housing located in a good neighbourhood (safe, close to family, friends and amenities) was identified as important (Carter et al., 2009; Ziersch et al., 2017a, 2017b). Likewise, housing satisfaction and neighbourhood satisfaction were significantly associated, highlighting the interlinkages between these two elements. Similar considerations have been observed in non-refugee populations—where people may develop ‘neighbourhood careers’, alongside housing-specific pathways (Clark et al., 2006). Interestingly, none of the housing features such as type of tenancy, overcrowding and time in housing were associated with housing satisfaction, giving weight to the idea that people prioritise location over and above other factors. However, it should be noted that the lack of significant findings for housing features may also relate to the ways that factors such as crowding are measured—which reflect ‘western’ ideas of household composition, rather than circumstances of multigenerational households which are more common in some cultures. While housing and neighbourhood satisfaction were both individually associated with overall resettlement satisfaction, when housing and neighbourhood satisfaction were considered together as predictors of resettlement satisfaction, only neighbourhood satisfaction remained significant, further suggesting a key aspect of resettlement satisfaction relates to the neighbourhood where housing is, rather than housing itself.

Notably, three demographic variables were consistently associated with housing, neighbourhood and overall satisfaction levels—region of origin, visa status and financial satisfaction. The findings in relation to financial precarity highlights the impact of financial issues on resettlement and integration outcomes, found in other research (Allsopp et al., 2014; Netto, 2011; Sampson & Gifford, 2010). While there are papers that examine experiences of specific groups in particular migratory contexts, there has been less examination of different region of origin variations in the same context, as was considered here. Potential reasons for country/region of origin variations might include experiences of different housing systems and housing quality in country of origin, differences in network breadth to assist with accessing housing and segmented discrimination, as well as some of the potential cultural factors associated with socialising and neighbourhood relations highlighted above. There are important avenues for future research in further understanding such potential cultural impacts on housing and neighbourhood satisfaction, as well as resettlement satisfaction more generally.

The higher housing, neighbourhood and overall satisfaction of those on permanent visas reflects the broader literature showing that asylum seekers face additional difficulties in the housing market and other features of resettlement (Hebbani et al., 2017; Hiebert et al., 2005; Murdie, 2008; Phillips, 2006; Robinson et al., 2007; Rose & Ray, 2001; Teixeira, 2006; Ziersch et al., 2017a, 2017b). Importantly, visa status was significant in predicting resettlement satisfaction at a multivariate level, illustrating that visa status had an impact on resettlement satisfaction beyond potential impacts on employment and financial status factors that may relate to visa conditions (e.g., work rights and access to government benefits).

### Strengths and Limitations

The detailed quantitative data that formed the basis of this study enabled a wide-ranging assessment of housing and neighbourhood experiences for refugees and also asylum seekers about which less is known. However, the sample was a convenience one. There is no existing sampling frame for refugees and asylum seekers over the last 10 years in Australia, and while the relative proportions of region of origin of the sample and the generally younger age groups reflect the focus of refugee resettlement in Australia, the findings may not be more broadly generalisable. The study was focused in the urban area of Adelaide in South Australia. Given that housing stock and market conditions often differ in regional areas (Beer, 2001; Costello, 2009), the findings may not reflect the experiences of asylum seekers and refugees in regional areas. Given that Australia has a strong regional resettlement program, this is an important area for future research.

In addition, in order to ensure external validity and cultural appropriateness (based on reference group feedback and survey piloting), the survey did not use existing measures—and indeed none exist for much of what this research aimed to explore. As such, response categories for questions such as what is important in housing and neighbourhood, and housing and neighbourhood problems, may not have been exhaustive and may have missed other factors. As an example of this, a larger list of housing problems was provided than neighbourhood problems which may have omitted important other neighbourhood issues or primed people to be less satisfied with their current housing. Resettlement satisfaction was measured with only one question. While resettlement satisfaction can to some extent reflect the extent of integration, this does not adequately reflect the two-way aspect of integration (for example, receiving community experiences were not considered in this study). Also, it is possible that someone could be satisfied with their resettlement experiences, but not be ‘integrated’ according to all the indicators highlighted in Ager & Strang’s, (2008) model. Within the survey format, we did not ask about conceptualisations of neighbourhood. While companion qualitative research did explore this in depth, we therefore do not have this detailed information for the survey participants.

### Theorising Neighbourhood in Integration

Notwithstanding these limitations, taken together, this analysis indicates that housing and neighbourhood are important for resettlement—those who were happier with their housing and neighbourhood were also happier overall with their resettlement—and therefore potentially integration. Other elements of the findings tie in with aspects of Ager and Strang’s model of integration. The significance of visa status also highlights what Ager and Strang refer to as the ‘foundation’ of integration—rights and citizenship and aspects of ‘facilitators’ of integration—stability (migration status). For asylum seekers with constrained rights and eligibility for government supports and no access to citizenship as a result of government policy (a ‘manufactured precarity’ (van Kooy & Bowman, 2019)), this shakier foundation was associated with lower satisfaction with housing and neighbourhood and overall resettlement. The significance of social connections to successful resettlement and integration, as outlined by Ager & Strang, 2008, was reflected in this study, through relationships with neighbours constituting an important aspect of neighbourhood and housing.

The findings suggest the importance of extending these aspects of integration to consider more carefully the role of neighbourhood in integration. Notwithstanding the limitations of the quantitative settlement satisfaction proxy for integration, our companion qualitative work Ziersch et al., 2017b and a broader literature highlights the importance of housing and neighbourhood for ontological security and integration (Easthope, 2004; Hiscock et al., 2001; Mallett, 2004). Taken together, the findings highlight the importance of neighbourhood in terms of social integration (Hynie et al., 2016)—where community welcome (positive attitudes and beliefs towards refugees) and institutional adjustments (neighbourhood level agencies and institutions) help shape experiences of refugees, and the two-way aspect of integration is important (Phillimore, 2020). Neighbourhoods may also be an important aspect of integration in their own right—where being in a ‘desired’ neighbourhood known to be safe and well-resourced may be a means and marker, in Ager and Strang’s terms, of integration as well as a means to achieving other elements of integration.

### Policy and Practice Implications

The findings also point to a range of policy implications and build upon other research/practice recommendations to improve housing and neighbourhood experiences of refuges and asylum seekers (Flatau et al., 2015; Rose, 2019). Efforts to improve financial security could include affordable housing schemes with eligibility for all visa holders (including those on temporary visas), and increased public housing availability, as well as full access to welfare payments for all visa holders and income-generating activities such as pathways to employment. Refugees may also need longer time in government supported housing as part of their initial resettlement to ensure that they are able to independently navigate the housing market, given that the majority will go into private rental properties. Issues with housing condition and features such as heating and cooling also speak to the need for minimum standards for housing. The issues identified with finding housing in the first place (once initial housing support had finished) highlights the importance of service provider support in securing subsequent housing (e.g., assistance with references, education in tenant rights and responsibilities), as well as facilitating social networks for refugees given the important role that networks appeared to be playing in securing housing. More generally, given the negative association between visa status and settlement and other research highlighting the detrimental impact of temporary visas on integration and wellbeing more broadly (Newman et al., 2019; Nickerson et al., 2019; Walsh et al., 2022; Ziersch et al., 2021), temporary visas must be abolished, and holders transferred to permanent protection visas. The incoming Australian government was elected on a promise to do this, but this change is yet to happen.

The study highlights the major role of neighbourhood location and safety in particular—the importance of this for ontological security and broader integration was highlighted above. Therefore, matching people to neighbourhood preferences in initial housing is important, including paying attention to proximity to social networks and services, as well as helping people to secure housing in their desired neighbourhood once the need to move to independent housing. This may be considered by some service providers but needs to be an explicit policy. Importantly, the high mobility of refugees may mean that they experience a range of neighbourhoods in their settlement journey and helping people to navigate neighbourhood transitions would also be supportive of integration. In addition, community development approaches to facilitating neighbourhood connections and welcome reflects a two-way approach to integration. In Australia, Welcoming Australia’s Welcoming City initiative is an example of this—where local councils become accredited for their efforts to be inclusive and welcoming. More generally, feeding priorities of refugees themselves into housing and neighbourhood policies are crucial to the institutional adaptation aspect of integration.

## Conclusion

This paper highlights the importance of positive housing and neighbourhood experiences for refugees and asylum seekers resettling in new countries, as well as the imperative to include a focus on neighbourhood in conceptualisations of integration. This is particularly important for refugees and asylum seekers, given threats to ontological security associated with forced migration (Carter et al., 2009; Easthope et al., 2018; Fozdar & Hartley, 2014; Phillips, 2006). Policy features help shape these experiences, particularly those related to temporary visas that limit financial and employment opportunities. While the contextual aspects of the Australian housing market and immigration and welfare policy are specific, the research highlights the broader potential impacts of policies on refugee and asylum seeker resettlement experiences and integration, in particular, the greater vulnerabilities of asylum seekers. Importantly, the research particularly indicates that neighbourhood, alongside housing, is an important policy consideration for resettlement countries, where refugees and asylum seekers would ideally be placed near their family, friends and ethnic communities, in areas where they feel safe and can develop a sense of belonging in order to rebuild a sense of ontological security. In summary, ensuring neighbourhood and contextual elements are included in understandings of integration will help develop more complete understandings of integration processes and lead to the best chance for successful outcomes for both new arrivals and receiving communities.

## Footnotes

1 Refugees are defined as people who meet the criteria for refugee status according to the United Nations High Commissioner for Refugees (UNHCR) and asylum seekers are defined as those awaiting their claims to refugee status or for other types of protection to be determined UNHCR. (2020). *What is a refugee?* https://www.unrefugees.org/refugee-facts/what-is-a-refugee/, but at times by criteria outlined by specific countries Türk, V., Edwards, A., & Wouters, C. (2017). *In Flight from Conflict and Violence: UNHCR's Consultations on Refugee Status and Other Forms of International Protection*. Cambridge University Press. In this paper, we use ‘refugee’ and ‘asylum seeker’ for brevity but acknowledge that this is only one aspect of identity.
